# Advancing food security and proposing future strategies: A comparative performance analysis of Global Food Security Index and Annual Population Changes of top five populous countries

**DOI:** 10.1371/journal.pone.0324231

**Published:** 2025-05-21

**Authors:** Xiuling Guo, Muhammad Islam

**Affiliations:** 1 Henan Police College, Zhengzhou, Henan, China; 2 Director of Agriculture (Statistics), Crop Reporting Service, Punjab, Pakistan; Arizona State University, UNITED STATES OF AMERICA

## Abstract

Rising global food insecurity driven by population growth needs urgent measure for universal access to food. This research employs Comparative Performance Analysis (CPA) to evaluate the Global Food Security Index (GFSI), its components [Affordability (AF), Availability (AV), Quality & Safety (Q&S) and Sustainability & Adaptation (S&A)] in tandem with Annual Population Change (APC) for world’s five most populous countries (India, China, USA, Indonesia and Pakistan) using dataset spanning from 2012 to 2022. CPA is applied using descriptive analysis, correlation analysis, Rule of Thumb (RoT) and testing of hypothesis etc. RoT is used with a new analytical approach by applying the significance measures for correlation coefficients. The study suggests that India should enhance its GFSI rank by addressing AF and mitigating the adverse effects of APC on GFSI with a particular focus on Q&S and S&A. China needs to reduce the impact of APC on GFSI by prioritizing AV and S&A. The USA is managing its GFSI well, but focused efforts are still required to reduce APC’s impact on Q&S and S&A. Indonesia should improve across all sectors with a particular focus on APC reduction and mitigating its adverse effects on AF, AV, and S&A. Pakistan should intensify efforts to boost its rank and enhance all sectors with reducing APC. There is statistically significant and negative relation between GFSI and APC for China, Indonesia and found insignificant for others countries. This study holds promise for providing crucial policy recommendations to enhance food security by tackling its underlying factors.

## Introduction

### Historical context and indicators of Global Food Security Scale

The World Food Summit in 1996 defined food security as a fundamental aspect of human well-being which includes the availability, access, utilization and stability of food resources [1–3]. The early 21^st^ century witnessed a surge interest in analyzing and evaluating global food security driven by growing population, environmental challenges, socio-economic disparities and political instability across the world [4–6]. This prompted the creation of Global Food Security Index (GFSI) to address the emerging challenges of global food securities. The GFSI is a tool used to assess and compare the food security situation of various countries and regions around the world [7,8]. GFSI is a vital instrument in today’s world. GFSI is developed in 2012 by research and analysis division of Economist Group called Economist Intelligence Unit (EIU) in collaboration with Barilla Center for Food & Nutrition (BCFN) [9,10]. The EIU established in 1946 and headquartered in London, UK. EIU is the research arm of the Economist Group. It offers global economic forecasting, industry analysis and business intelligence etc. The Barilla Center for Food & Nutrition (BCFN) is a research center founded by the Barilla Group, an Italian multinational food company. Established in 2009, BCFN focuses on global food security, nutrition, sustainability and the future of food. The center is involved in creating strategies for sustainable food systems that balance the health and sustainable agricultural practices. GFSI is updated annually by EIU and it emerges as a comprehensive and influential tool providing insights into the complex and multifaceted issue of global food security concerns for 113 countries across the world. The GFSI is aligned with the United Nations’ Sustainable Development Goal (Zero Hunger) which calls for an end to hunger, improved nutrition and sustainable agriculture by 2030 [11,12]. The GFSI provides a way to track progress toward this goal and identify areas in the world where action is needed [12,13].

GFSI utilizes a meticulous and data-driven approach by consolidating a wide range of indicators and sub-indicators that capture the multifaceted aspects of food security. GFSI scores range from 0 to 100 and higher scores indicating better performance [14,15]. GFSI combines a set of indicators each of which can be seen as elements contributing to food security [16,17]. It incorporates a combination of indicators each of which can be considered as factors contributing to food security [8,18,19]. GFSI aims to offer insights into a country’s relative level of food security compared to others countries and it assess food security status based on affordability, availability, quality & safety, and sustainability & adaptation [20,21].

### Global perspective of GFSI

One of the most compelling aspects of the GFSI is its truly global perspective. GFSI ranks countries and regions on their food security performance by highlighting disparities and providing valuable insights into the geographic distribution of this critical issue [22–24]. This global outlook enables policymakers, researchers and stakeholders to identify regions and countries where food security is most precarious and to need tailor interventions accordingly [25]. The GFSI not only serves as a diagnostic tool but also has profound policy implications [20,26]. Božić and Nikolić [27] reported countries with lower GFSI scores can use the index to identify areas of weakness and develop strategies to improve food security. Conversely, those with higher scores can examine their policies and practices as potential models for others. Nations can work toward strengthening their food security systems by analyzing the factors that contribute to high or low scores. GFSI adapts to address evolving challenges such as changes in the global landscape, climatic variations, globalization, and technological advancements [28,29]. Its ongoing development reflects the commitment to addressing the most pressing issues in global food security and making it a dynamic tool.

### Population growth and GFSI in the world’s top five most populous nations

The Population Division of United Nations Department of Economic and Social Affairs (UN-DESA) through the World Population Prospects 2022 offers the global community up-to-date and readily available information on population statistics, in-depth analysis of demographic patterns and developmental results across every nation and region worldwide [30,31]. According to United Nations projections, the actual annual increase in the global human population is approximately 83 million with a growth rate of 1.1% and this trend is expected to persist with an estimated total population of 8.6 billion by mid-2030, 9.8 billion by mid-2050, and 11.2 billion by the year 2100 [32,33]. The GFSI is a valuable tool for assessing a nation’s food security. It considers factors i.e. affordability (AF), availability (AV), quality & safety (Q&S), and sustainability & adaptation (S&A) of food to provide a comprehensive picture of the state of food security in a given country. Worldometer’s Population Page provides real time global population estimates based on UN data and advanced models and it is a valuable source for researchers, policymakers and analysts in fields like food security, urban development and sustainability [34–38]. The silent features of population as presented in World Population on worldometers available at https://www.worldometers.info/population/ and GFSI of five top populous countries available at https://impact.economist.com/sustainability/project/food-security-index/ are described below. India is the most populous country in the world and tracked to surpass China. India’s estimated population lies about 1428.63 million and contributed 17.76% share in total world population with average growth rate of 0.91%. India is ranked 68^th^ on the GFSI 2022 with 80^th^ for affordability, 42^th^ for availability, 67^th^ for quality & safety and 71^th^ for sustainability & adaptation. China is 2^nd^ most populous country in the world with population of 1425.67 million. China’s population continues to grow steadily. The government has implemented the “one-child policy” which has slowed the growth rate. China contributed to 17.72% share in total world population. 65.0% of China’ population is urban. China is ranked 25^th^ on the GFSI 2022 with 33^th^ for affordability, 2^nd^ for availability, 46^th^ for quality & safety, and 55^th^ for sustainability & adaptation. The USA has a lower population density compared to some other countries on the list and still faces population growth driven by immigration and natural increase. The USA’s population is estimated at 340.00 million and sharing to 4.23% of the total world population. USA ranks 3^rd^ at most populous country in the world. The 82.9% of the USA’s population is urban. USA stood on 13^th^ on the GFSI 2022 with 29^th^ for affordability, 31^th^ for availability, 3^th^ for quality & safety and 12^th^ for sustainability & adaptation. Indonesia is the most populous nation in Southeast Asia. It ranks 4^th^ at most populous country in the world with growth rate of 0.82%. Indonesia’s 2023 population is estimated at 277.53 million and sharing equivalent to 3.45% of the total world population. The 59.1% of the Indonesia’s population is urban. Indonesia lies at 63^th^ on GFSI 2022 with 44^th^ for affordability, 84^th^ for availability, 78^th^ for quality & safety and 83^th^ for sustainability & adaptation. Pakistan’s population growth is very high in Asian countries and it faces the dual challenge of providing food security and improving the quality of life for its citizens. This requires investments in agriculture, infrastructure and education. Pakistan’s 2023 population is estimated at 240.49 million and sharing equivalent to 2.99% of the total world population. Pakistan ranks 5^th^ in world most populous countries with average growth rate of 1.74%. The 34.7% of Pakistan’s population is urban. Pakistan ranks on 84^th^ on GFSI 2022 with 75^th^ for affordability, 61^th^ for availability, 97^th^ for quality & safety and 106^th^ for sustainability & adaptation.

## Literature review

The importance of food security has grown significantly over the last decade and more it has been recognized as an essential and crucial element of national security and food sustainability [39–41]. Preventing global food scarcity, boosting crop yields and continued technological development have become critical concerns in agriculture and food security [42–45]. Ammar *et al.* [21] reported population growth can have significant impacts on a country’s food security and this impact is reflected in the GFSI. In similar way How *et al.* [46], Estrada *et al.* [47], Guo *et al.* [48] emphasized that population growth can affect the GFSI in term of increasing food demand, straining food supply chain, overexploitation, degradation of natural resources, rising food prices, policies governance and making it more challenging for a country to ensure food is available to meet the needs of its citizens [44,49–53]. They also reported that GFSI is negatively impacted if a country’s food production and distribution systems are not adequately prepared for its increasing demand. It is important to note that the relationship between population growth and the GFSI is complex and the specific impact can vary from country to country. The GFSI considers multiple factors and population growth is just one of them. Hatab *et al.* [54] reported that countries often need to focus on sustainable agricultural practices, resource management, infrastructure development, socio-economic and political stability over the long period of time to improve its food security in the face of population growth. Chen *et al.* [18] conducted research on the GFSI utilizing a hierarchical data envelopment analysis method to create a multi-faceted indicator. This study revealed that addressing food availability should be the foremost policy focus in low to medium income countries where food shortages are most pronounced. These findings offer valuable insights to enhance the planning and implementation of global food security initiatives. Sukereman *et al.* [55] reported national food security is a paramount goal for governments and researchers. They compared food security levels in Malaysia (an upper-middle-income country), Singapore (a high-income country) and Indonesia (a lower-middle-income country) using GFSI i.e. affordability, availability, quality& safety, natural resources and resilience from 2012 to 2020. The results revealed that food security trends from 2012 to 2020 was not linear and influenced by various factors. High-income countries exhibit greater food security followed by upper-middle and lower-middle-income countries. Collaborative efforts among these nations are needed to address weaknesses, leverage strengths, bridged food security gaps and aligned with the sustainable development. Thomas *et al.* [15] studied the GFSI’s framework and reported its contention for the weighting criterion of GFSI for 113 countries and recommended to use the GFSI with other indicator of food insecurity, food consumption and nutritional status of populations. Izraelov and Silber [8] highlighted the consequences of food insecurity by exploring the food security indicators of GFSI. They found that GFSI weights and indicators provided a reasonable ranking of countries for food security levels. Stezhko [56] reported vulnerability in terms of food security by considering financial and physical accessibility, food quality and safety using GFSI. It is found that nations with low GFSI scores exhibit low food accessibility but have moderate indicators for food availability, quality and safety. Pyzhikova *et al.* [16] analyzed GFSI in Europe, Africa, and America etc. and reported that high food security in Europe and America while African states experienced chronic malnutrition and hunger due to population pressure and food disparity. Miladinov [57] analyzed the effect of rural and urban population growth, along with GDP per capita, on undernourishment in low and middle income countries using World Bank data from 2001 to 2020 and econometric models. The findings show that growth in rural populations contributes to higher levels of undernourishment, particularly in upper middle income countries, while urban population growth tends to reduce undernourishment. Long-term trends in population dynamics play a significant role in shaping undernourishment rates. The study’s findings provide significant policy implications regarding the effects of rural and urban population growth and GDP per capita on undernourishment. This is valuable for academic research as well as for governments, policymakers and the public in identifying and prioritizing key food security factors. Food and Agricultural Organization (FAO) and the International Food Policy Research Institute (IFPRI) voice profound apprehension and urging the enhancement of policies for promoting food sustainability and food security [10,24,40,58,59]. Carvalho [60] reported that the global population growth rate is projected to be 1.2%, and six countries i.e. China, India, Pakistan, Indonesia, Bangladesh and Nigeria will collectively responsible for half of this growth. Sadigov [61] reported global population is expected to surpass 8.5 billion by 2030, reach 9.7 billion by 2050 and further increase to 11.1 billion by the year 2100. In addition to this situation Islam et al. [19] and Falcon *et al*. [62] reported that food demand will raise up to 70% in developed counters and it would be 100% for the developing countries. Accurately evaluating food security at global neck is essential for planning. Patel and Sharma [63], Puri [64], Sinha [65] and Barman [66] addressed that food availability, nutritional needs and hunger for the expanding population posed a significant challenge for the Indian government. Various studies have developed considering Global Food Security Index (GFSI) as a standardized framework and enabling an examination of worldwide food systems’ dynamics to comprehend the root causes of food insecurity using different socio-economic parameter, demographical signs, health characteristics and crop production etc. [48,67–70]. This studied differentiate these studies and directly relate to the emphasized of GFSI components and population effects on the food security using the GFSI score of five most populous countries in the world.

### Research contribution to the demographic literature

This study makes a valuable contribution to the demographic literature by exploring the changing relationship between population growth and food security, using the Global Food Security Index (GFSI) as a key reference. It examines how demographic trends impact food demand and the ability of national systems to maintain sustainable food availability, accessibility and stability. The study extends theories such as Malthusian Theory, which argues that unchecked population growth can lead to food scarcity [71] and Demographic Transition Theory, which suggests that industrialization moderates population growth and shifts the focus of food security from production to distribution, policy and access [72–74]. By integrating systems theory and sustainable development theory, it recognizes food security as a multifaceted issue influenced by governance, technology, economic development and environmental sustainability [75]. This research integrates demographic theories with empirical food security analysis offering insights into how population pressures affect food production and distribution systems. By analyzing population data alongside food security measures, it provides a modern interpretation of classical demographic theories and offered valuable empirical evidence for policymakers. The study also addresses the ongoing debate in Malthusian theory regarding whether population growth hinders or drives improvements in food systems [76] and it enhances scientific understanding of how demographic pressures influence national food security with implications for the Sustainable Development Goals (SDGs), especially SDG 2: Zero Hunger [77–79].

### Problem statement, Novelty and objective of the study

#### Problem statement.

The rapid population growth in the five most populous countries presents a significant challenge to global food security. Analysis of GFSI reveals that while each of these nations employs distinct strategies to ensure food security, they also face critical weaknesses based on key GFSI indicators. The disproportionate increase in population, if not accompanied by a corresponding rise in agricultural productivity, exacerbates food resource constraints, particularly affecting low income populations’ access to adequate nutrition. As global population trends continue to rise, understanding the food security dynamics of these highly populated nations is crucial for addressing the broader implications of food insecurity for well versed policy making and sustainable development.

#### Novelty and objectives of the study.

Despite extensive research on food security and population dynamics, a comprehensive comparative performance analysis (CPA) of the GFSI and its key components, i.e., AF, AV, Q&S, and S&A in relation to annual population change (APC) in the top five most populous countries has not been conducted using statistical tools. This study fills that gap by employing a rigorous statistical approach, offering new insights into the interplay between population growth and food security indicators. Additionally, this study introduces a novel analytical perspective by incorporating the Rule of Thumb (RoT) for correlation coefficients, providing a unique methodological contribution to food security research. The uniqueness of this approach combined with its focus on these specific countries enhances the study’s contribution to offer country specific insights and policy recommendations systematically by evaluating the GFSI and APC trends. This study is enabling data driven decision making for improved food security strategies.

This study aims to generate actionable insights that will assist policymakers, researchers and stakeholders in designing effective food security strategies in response to population dynamics. Specifically, it seeks to analyze and compare the performance of the overall GFSI and its key components (AF, AV, Q&S, and S&A) in the five most populous countries. This study examines the impact of APC on the GFSI and its components, utilizing statistical tools, including the Rule of Thumb for correlation coefficients, to identify patterns and trends in food security performance across these nations. It aims to explore the relationship between population growth and food security, identifying underlying trends that may influence future policy decisions. This study will propose strategic policy recommendations to enhance food security while addressing the challenges, if posed by population growth.

### Data and methods

Macrotrends is a financial and economic research platform based in the United States, providing historical data, charts, and analysis for researchers and economists. The population data on Macrotrends is sourced from the United Nations, World Population Prospects (WPP), maintained by the UN Department of Economic and Social Affairs (UNDESA), Population Division [80–83]. Macrotrends presents these official UN datasets in a user friendly format for trend analysis and research. It covers a wide range of data including population size, growth rates, age distribution, fertility rates, mortality rates and migration patterns. The data is presented and allowing for world wise analysis and comparisons. Annual Population Changes (APC) data is collected from the Macrotrends for five most populous countries in the world comprises from 2012 to 2022 available at https://www.macrotrends.net/global-metrics/countries/ranking/population. The GFSI is a tool developed by the research and analysis division of the Economist Group (EG) called Economist Intelligence Unit (EIU), in collaboration with the Barilla Center for Food & Nutrition (BCFN) and it measure the rank and scores of food security on a global scale [9,10]. The Economist Intelligence Unit (EIU) founded in 1946 and based in London, UK, serves as the research division of The Economist Group. EIU provides global economic forecasts, industry analysis and business intelligence. TheBCFN established in 2009 by the Barilla Group, an Italian multinational food company, is dedicated to addressing global food security, nutrition, sustainability and the future of food. The center focuses on developing strategies for sustainable food systems that promote both health and sustainable agricultural practices. The GFSI index considers various factors to provide a comprehensive understanding of the state of food security in different countries. These factors include Affordability (AF), Availability (AV), Quality & Safety (Q&S) and Sustainability & Adaptation (S&A). Data regarding GFSI is collected from Global Food Security Index datasets for the year 2012 to 2022 available at https://impact.economist.com/sustainability/project/food-security-index/. This presented the GFSI scores and make its GFSI rank for the 113 countries around the world. GFSI scores are normalized 0–100 and 100 stands for the best score & vice versa. The GFSI also ranks the countries and 1 represents the highest (best) rank.

### Comparative Performance Analysis (CPA)

This study provides the comparative performance analysis (CPA) of GFSI scores and APC along with their corresponding GFSI components i.e. AF, AV, Q&S and S&A for top five most populous countries of the world using different statistical approaches to extract and to compare the meaning full information inside from data. This study lead to layout the direction oriented policies for food insecurity threats for these countries. This analysis identifies improvements or deteriorations in performance over the specified period. GFSI index and its components are used to compare the significance and basic statistics. The tabulation is presented to elaborate the basic statistics. Correlations coefficients are applied to check the significance of the components against the overall score of GFSI and APC. SPSS and Python’s key library Scikit-learn are used to analyze and to compare the results.

### Descriptive CPA of GFSI and APC

The CPA is conducted using the tabulation to assess the change in scores (%) and the corresponding change in ranks for the year 2022 in comparison with 2012.

### CPA using correlation and testing of hypothesis

Correlation analysis (r) is employed to evaluate the strength and direction of the association between variables and to quantify the extent of their linear relationship, while hypothesis testing is conducted to assess the statistical significance of the observed correlation [84–87]. The research aims to unveil the interdependence and potential relationships between GFSI and APC using correlation analysis and testing of hypothesis to measure the statistical significance of correlation coefficients.


$$\begin{array}{c} Correlation\;(r)\;=\;\frac{\sum (Y_{1}-{\overset{―}{Y}}_{1})\;(Y_{2}-{\overset{―}{Y}}_{2})}{\sqrt{[\sum \left({Y_{1}-{\overset{―}{Y}}_{1})}^{2}\right]\times [\sum {\left(Y_{2}-{\overset{―}{Y}}_{2}\right)}^{2}]}} \end{array}$$ (1)

Where $"{\overset{―}{Y}}_{1}"\;$stands the mean of variables $"Y_{1}"$ and $"{\overset{―}{Y}}_{2}"\;$stands the mean of variable$"Y_{2}"$. The following statistical hypothesis is formulated to assess the significance of correlation coefficients at 5% level of significance.

$H_{1}$: Correlation coefficient found statistical significant (s) for GFSI & GFSI components.

$H_{2}$: Correlation coefficient found statistical significant (s) for APC & GFSI components.

$H_{3}$**:** Correlation coefficient found statistical significant (s) for GFSI & APC

### Rule of Thumb for correlation coefficients (RoT) and test of significance

The correlation between the two variables is represented by a quantified value ranging from -1 to +1. A value of zero indicates no correlation while ±1 signifies a complete perfect/negative correlation. The strength of the correlation intensifies from 0 to ±1. The term “Rule of Thumb (RoT)” generally denotes a guiding principle or criterion frequently employed by researchers to interpret correlation coefficients and these criteria categorize the degree of correlation between two variables into groups such as negligible, lower, moderate, strong, and very strong [86,88–90]. RoT is used with a new analytical approach by applying the significance measures for correlation coefficients. RoT provides a clear and standardized method for interpreting correlation coefficients, ensuring consistency in analysis. By categorizing correlations based on their strength while considering statistical significance, RoT improves result clarity and facilitates meaningful interpretation. This approach helps to differentiate true correlations from random occurrences and leading to more accurate assessments of statistical relationships. Ultimately, it enhances the reliability of research findings and supports for good policy decision. Table 1 describes the general guide of RoT to interpreting correlation coefficients with identification of statistical significance (S) or insignificance (I) of coefficients.

**Table 1:** RoT for correlation coefficients along with statistical significance.

Range	Category	Descriptions	Significance (S)	Insignificance (I)
0.00 to 0.20 (±)	Negligible (±)	Relationship between variables is nonexistent	Negligible significance (NS) (±)	Negligible insignificance (NI) (±)
0.20 to 0.40 (±)	Lower correlation (±)	Correlation is present, but it’s relatively lower	Lower significance (LS) (±)	Lower insignificance (LI) (±)
0.40 to 0.60 (±)	Moderate correlation (±)	Moderate correlation between variables	Moderate significance (MS) (±)	Moderate insignificance (MI) (±)
0.60 to 0.80 (±)	Strong correlation (±)	Relationship between variables is strong	Strong significance (SS) (±)	Strong insignificance (SI) (±)
0.80 to 1.0 (±)	Very strong correlation (±)	Variables are highly correlated	Very strong significance (VSS) (±)	Very strong insignificance (VSI) (±)

## Results and analysis

**Fig 1:**
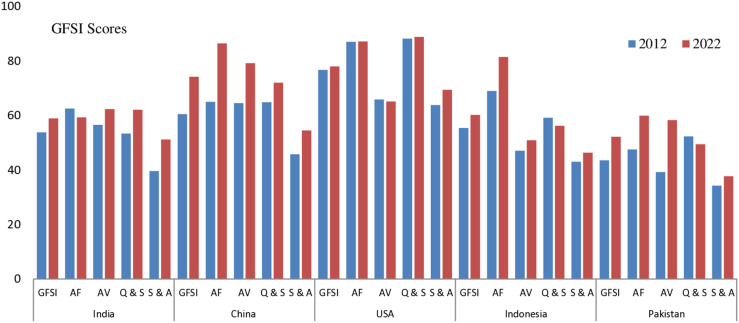
Comparative performance analysis of GFSI scores for the years 2012 and 2022.

The GFSI scores of the top five most populous countries offer valuable insights into the advancements and challenges in global food security over the past decade. This analysis explores changes in overall GFSI scores alongside their key determinants to identify emerging trends, disparities and policy implications shaping food security dynamics. Table 2 and Fig 1 provides the CPA on selected countries’ GFSI scores for the years 2012 and 2022 with their corresponding GFSI scores for its determinants i.e. AF, AV, Q&S and S&A. The table also includes the APC for top five most populous countries of the world. It is depicts that overall GFSI score found 58.9, 74.2, 78.0, 60.2 and 52.2 for 2022 while it was 53.8, 60.5, 76.7, 55.4 and 43.5 for 2012, respectively for India, China, United States, Indonesia and Pakistan. GFSI found good for USA and China followed by Indonesia, India and Pakistan.

**Table 2:** Comparative performance analysis of GFSI and its determinates scores/ranks with population growth rate.

Countries		Years	GFSI	AF	AV	Q & S	S & A	APC
India	Scores	2012	53.8	62.5	56.5	53.4	39.6	1.34
		2022	58.9	59.3	62.3	62.1	51.2	0.68
		Change (%) in score	9.48	-5.12	10.27	16.29	29.29	-49.25
	Ranks	2022	68	80	42	67	71	–
		Change in 2022 compared with 2012 ▲ = Rank improved ▼ = Rank deteriorated	▼1	▼10	▼3	▲8	▲10	–
China	Scores	2012	60.5	65	64.5	64.8	45.8	0.7
		2022	74.2	86.4	79.2	72	54.5	0
		Change (%) in score	22.64	32.92	22.79	11.11	19.00	-100.0
	Ranks	2022	25	33	2	46	55	–
		Change in 2022 compared with 2012 ▲ = Rank improved ▼ = Rank deteriorated	▲24	▲32	▲21	▲7	▼11	–
USA	Scores	2012	76.7	87	65.8	88.2	63.8	0.88
		2022	78	87.1	65.1	88.8	69.4	0.38
		Change (%) in score	1.69	0.11	-1.06	0.68	8.78	-56.82
	Ranks	2022	13	29	31	3	12	–
		Change in 2022 compared with 2012 ▲ = Rank improved ▼ = Rank deteriorated	▼8	▼12	▼15	▼2	▼8	–
Indonesia	Scores	2012	55.4	69	47	59.1	43	1.26
		2022	60.2	81.4	50.9	56.2	46.3	0.64
		Change (%) in score	8.66	17.97	8.30	-4.91	7.67	-49.21
	Ranks	2022	63	44	84	78	83	–
		Change in 2022 compared with 2012 ▲ = Rank improved ▼ = Rank deteriorated	▼1	▲17	▼14	▼13	▼25	–
Pakistan	Scores	2012	43.5	47.5	39.2	52.3	34.2	1.81
		2022	52.2	59.9	58.3	49.4	37.7	1.91
		Change (%) in score	20	26.11	48.72	-5.54	10.23	5.52
	Ranks	2022	84	75	61	97	106	–
		Change in 2022 compared with 2012 ▲ = Rank improved ▼ = Rank deteriorated	▲10	▲12	▲35	▼18	▼4	–

1. GFSI 2022. Scores are normalized 0–100, where 100 = best score & vice versa.

2. GFSI 2022. Ranks for countries, where 1 = best rank & vice versa.

### India

By 2022, India’s GFSI score increased to 58.9 in 2022 compared 53.8 in 2012 and showing a 9.48% improvement. Significant improvements seen in AV score (10.27%), Q&S (16.29%) and S&A score (29.29%) while decay observed for AF score (-5.12%). The APC decreased from 1.34% in 2012 to 0.68% in 2022 and this depicts a decrease of -49.25%. The CPA of GFSI rank in 2022 compared with 2012 show that overall rank deteriorated about 1, 10 and 3 for GFSI, AF and AV while improved 8 and 10 for Q&S and S&A.

### China

In 2012, China had a GFSI score of 60.5 and it is improved to 74.2 in 2022 depicting a 22.64% increase in GFSI score. It is GFSI rank stood at 25^th^ in 2022. China saw significant improvement in AF (32.92%), AV (22.79%), Q&S (11.11%) and S&A (19.0%). China shows good performance in APC reaching to 0% in 2022 (-100%). China’s rank deteriorated about 11 for S&A and it is improved 24, 32, 21 and 7 for GFSI, AF, AV and Q&S.

### USA

USA’s GFSI score found 76.7 in 2012 and slightly grow to 1.69% in 2022 with its score 78.0 in 2022. Similarly slight change observed for AF about 0.11%, AV about -1.06%, Q&S about 0.68% and good change for S&A about 8.78%. As we seen the rank of USA, it is observed to deteriorated to 8 for GFSI, 12 for AF, 15 for AV, 2 for Q&S and 8 for S&A. APC reported -56.82% decreased in 2022 compared with 2012. USA ranked at 13^th^ GFSI ranks 2022.

### Indonesia

Indonesia reported a GFSI score of 55.4 in 2012 and it is improved to 60.2 in 2022. The GFSI score improved 8.66%. Indonesia improved their score 69.0 to 81.4 for AF (17.97%), AV from 47 to 50.9 (8.30%) and S&A from 43.0 to 46.3% (7.67%) while for Q&S decreased from 59.1 to 56.2 (-4.91%) in 2022 comparing with 2012. The APC decreased about -49.21% in 2022. Indonesia’s rank deteriorated 1, 14, 13 and 25 in GFSI, AV, Q&S and S&A while its rank improved to 17 for AF in 2022 comparing with 2012.

### Pakistan

By 2022, Pakistan’s GFSI score witnesses 52.2 compared with 43.5 in 2012 and showing the increase of 20.0%. Significant improvements is seen in scores of AF from 47.5 to 59.9 (26.11%), AV from 39.2 to 58.3 (48.72%) and S&A from 34.2 to 37.7 (10.23%) while for Q&S it is decreased to 49.4 from 52.3 (-5.54%). The APC increased to 1.91% in 2022 from 1.81% in 2012 and showing the increase of 5.52%. The CPA of GFSI rank in 2022 compared with 2012 show that overall rank deteriorated about rank 18 and 4 for Q&S and S&A while found improved to 10, 12 and 35 for GFSI, AF, and AV.

### Comparative performance analysis of GFSI and APC using correlation

**Fig 2:**
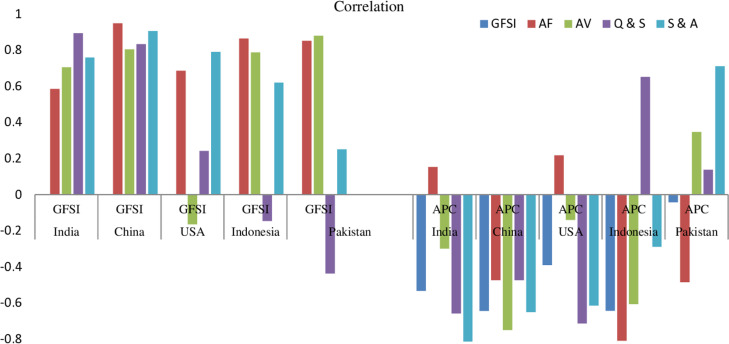
Comparative assessment of correlations between GFSI, GFSI’s components and APC.

Understanding the relationship between the GFSI and APC is crucial for assessing food security dynamics in the world’s most populous countries. This analysis explores the correlation between GFSI, its key components, and APC to evaluate how population growth is interlinked with food security. Table 3 and Fig 2 provide a detailed assessment of these correlations, highlighting statistical significance and trends across the five most populous nations.

**Table 3:** Correlations and significance of GFSI & annual population change (APC) against GFSI components.

	Parameters		GFSI	AF	AV	Q & S	S & A	APC
India	GFSI	Corr.	--	0.585	0.705*	0.894**	0.759**	-0.533
		Sig.	--	0.059	0.015	0.00	0.007	0.091
	APC	Corr.	-0.533	0.153	-0.299	-0.658*	-0.874**	--
		Sig.	0.091	0.654	0.371	0.028	0.000	--
China	GFSI	Corr.	--	0.949**	0.804**	0.833**	0.906**	-0.644*
		Sig.	--	0.000	0.003	0.001	0.000	0.032
	APC	Corr.	-0.644*	-0.474	-0.750**	-0.474	-0.651**	--
		Sig.	0.032	0.141	0.008	0.141	0.030	--
USA	GFSI	Corr.	--	0.686*	-0.164	0.242	0.791**	-0.39
		Sig.	--	0.02	0.629	0.474	0.004	0.235
	APC	Corr.	-0.39	0.218	-0.14	-0.714*	-0.615*	--
		Sig.	0.235	0.519	0.680	0.014	0.044	--
Indonesia	GFSI	Corr.	--	0.865**	0.788**	-0.146	0.620*	-0.643*
		Sig.	--	0.001	0.004	0.668	0.042	0.033
	APC	Corr.	-0.643*	-0.809**	-0.606*	0.652*	-0.289	--
		Sig.	0.033	0.003	0.048	0.030	0.389	--
Pakistan	GFSI	Corr.	--	0.852**	0.880**	-0.437	0.251	-0.042
		Sig.	--	0.001	0.000	0.178	0.456	0.903
	APC	Corr.	-0.042	-0.485	0.347	0.137	0.711*	--
		Sig.	0.903	0.130	0.295	0.688	0.014	--

#### India.

The GFSI values as an indicative of overall food security are examined in relation to AF, AV, Q&S and S&A. Significant positive correlation (Sig < 0.05) are observed for India with correlation coefficient 0.705 for AV, 0.894 for Q&S and 0.759 for S&A while positive insignificant (sig > 0.05) for AF with correlation 0.585. The APC found negative and insignificant (sig > 0.05) with correlation coefficient -0.533. The table also explores the relationship between APC and GFSI scores. Positive and insignificant (sig > 0.05) relation with a value of 0.153 is found for AF while negative and insignificant with a value of -0.299 is found for AV. There are negative and statistically significant (sig < 0.05) relations exist for Q&S with coefficient -0.658 and S&A with correlation coefficients as -0.874.

#### China.

China demonstrated strong positive and statistically significant (sig < 0.05) relation for GFSI with AF, AV, Q&S, and S&A with coefficients 0.949, 0.804, 0.833 and 0.906 while reported negative and significant for APC with coefficient -0.644. APC reflected negative and significant relations with GFSI, AV and S&A with coefficients -0.644, -0.750 and -0.651 while found negative and insignificant for AF and Q&S with coefficients as -0.474 for both components.

#### USA.

USA shows strong positive and statistically significant (sig < 0.05) relation for GFSI with AF, and S&A with coefficient 0.686 and 0.791, weak positive and insignificant for Q&S (0.242) while found insignificant and weak negative with coefficients -0.164 for AV. The APC found insignificant and adversely hitting the GFSI with coefficients as -0.39. APC reflected poor insignificant and negative relation with AV as -0.14 while strong negative and significant for Q&S as -0.714 and S&A as -0.615. APC found weak insignificant positive for AF as 0.218.

#### Indonesia.

Indonesia demonstrated strong positive and significant relation for GFSI scores with AF (0.865), AV (0.788) and S&A (0.620) while found negative and poor for Q&S as -0.146. APC is adversely and significantly hitting the GFSI with coefficient -0.643. APC yields significant and negative effects on AF (-0.809), AV (-0.606) and S&A (-0.289) while positive for Q&S with coefficient as 0.652.

#### Pakistan.

There are significant strong and positive relation exist for GFSI with AF and AV with coefficients as 0.852 and 0.880, weak and insignificant for S&A (0.251) while negative and insignificant for Q&S (-0.437). The APC found insignificant and negative with a coefficient value of -0.042. Pakistan reveals positive relation for APC with AV (0.347), Q&S (0.137) and S&A (0.711). APC found negative for AF with correlation coefficient as -0.485. All the GFSI’ determinates found insignificant except S&A when it is compared for APC.

### Rule of Thumb for correlation coefficients

The RoT is a commonly used guideline for interpreting correlation coefficients, helping researchers to classify relationships as negligible, low, moderate, strong, or very strong. While RoT was originally employed for basic correlation analysis, it is now being integrated with advanced analytical techniques that also consider significance measures for more complex data analysis. Table 4 presented the comparison of rule of thumb for correlation coefficients both for GFSI and APC against GFSI components. For GFSI, India shows the MI (+) relation for the AF, SS (+) relation with AV and S&A while it is found VSS (+) for Q&S. For APC, there is MI (-) relation with GFSI while negligible for AF, LI (-) for AV, SS (-) for Q&S and VSS (-) for S&A. China shows VSS (+) relation with all GFSI components. For the APC, China depicts SS(-) relation with GFSI, AV and S&A while it is found as MI(-) for AF and Q&S. USA revealed SS (+) relation for AF and for S&A while negligible for AV and LI (+) relation for Q&S. APC have LI (-) relation with GFSI in USA. APC have LI (+) relation with AF, negligible for AV and SS (-) relation for Q&S and S&A. Comparing GFSI, Indonesia depicts VSS (+) relation for AF, SS (+) relations for AV, negligible for Q&S, SS(+) relation for S&A and SS(-) for APC. For APC, Indonesia depicts VSS (-) relation for AF, SS (-) for AV, SS (+) for Q&S and LI (-) for S&A. Comparing GFSI, Pakistan stands with VSS (+) relation for AF and AV, MI (-) for Q&S, LI (+) for S&A and negligible negative APC. For APC, Pakistan stands MI (-) relation for AF, LI (+) for AV, negligible for Q&S and SS (+) for S&A.

**Table 4:** Rule of Thumb for correlation coefficients.

Countries	Parameters	GFSI	AF	AV	Q & S	S & A	APC
India	GFSI	--	MI (+)	SS(+)	VSS(+)	SS(+)	MI(-)
	APC	MI (-)	NI (+)	LI (-)	SS(-)	VSS (-)	---
China	GFSI	--	VSS (+)	VSS (+)	VSS(+)	VSS (+)	SS (-)
	APC	SS (-)	MI (-)	SS(-)	MI(-)	SS(-)	--
USA	GFSI	--	SS (+)	NI (-)	LI (+)	SS(+)	LI (-)
	APC	LI (-)	LI (+)	NI (-)	SS (-)	SS (-)	--
Indonesia	GFSI	--	VSS (+)	SS (+)	NI (-)	**SS (+)**	SS(-)
	APC	SS (-)	VSS(-)	SS (-)	SS (+)	LI (-)	--
Pakistan	GFSI	--	VSS (+)	VSS (+)	MI (-)	LI (+)	Ni (-)
	APC	NI (-)	MI (-)	LI (+)	NI (+)	SS(+)	--

## Discussions

This study aimed to evaluate the effects of population dynamics on food security using the Global Food Security Index (GFSI) framework, focusing on five of the most populous countries. The findings confirm the research hypothesis that rapid population growth significantly impacts food security and its components. The primary research question explored whether higher population pressure significantly affects the overall food security levels, as indicated by the GFSI scores. The central hypothesis posited a negative relationship between population size and food security due to increased demand on limited resources, consistent with Malthusian and Neo-Malthusian theoretical perspectives which suggest that unchecked population growth can outpace food production and availability [63,64]. These results align with the earlier works of Sinha [65] and Barman [66], who observed that demographic expansion continues to exacerbate food system vulnerabilities. Our study further contextualized these challenges within the GFSI framework by isolating the subcomponents of food availability, affordability, quality, safety, sustainability and adaptations, thus providing a structured understanding of where the gaps lie. The findings also resonate with previous empirical studies that highlighted socio-economic and demographic parameters as critical determinants of food security [48,68,70]. Countries such as the United States and China scored good on food affordability and quality, reinforcing that economic strength and policy infrastructure play significant roles in countering the negative impacts of population pressure. On the contrary, populous nations with moderate-to-low economic development struggle with issues such as food distribution inefficiencies, malnutrition, and low dietary diversity. The theoretical framework grounded in food systems analysis and GFSI conceptualization helped us interpret the findings more holistically. Ozkaya and Ozkaya [67], and Markina *et al.* [69] argued that food security must be viewed as a multi-dimensional construct and our study supports this by demonstrating how population stress affects each GFSI dimensions. This reinforces the importance of a disaggregated approach to food security policy and assessment. Our findings align with the theoretical perspectives and empirical evidence discussed in the literature review. For example, Padder *et al*. [42–44] and Taqui *et al*. [45] highlighted the importance of ongoing technological advancements and enhanced agricultural practices in addressing the pressure that increasing populations place on food systems. Our findings also support Ammar *et al.* [21], who reported that demographic pressure is directly reflected in GFSI fluctuations. The current analysis echoes the concerns raised by Guo *et al.* [48] and Wani *et al.* [25], who emphasized the multidimensional nature of food security. As population size increases, countries with fragile food production systems experience a disproportionate impact on food security. This aligns with the findings of Rather *et al.* [51–53] that underscore the vulnerability of food supply chains under population stress. The empirical findings also validate the complexity discussed by Hatab *et al.* [54], who argue that the relationship between population dynamics and GFSI is not linear and must be understood within a broader socio-economic and political framework. Countries with better infrastructure, governance and sustainable agricultural practices are more resilient to the adverse effects of demographic growth. Our study also integrates well with the conceptual model of Chen *et al.* [18], who proposed that addressing food availability should be the primary policy focus in low-to-middle-income countries. This is particularly relevant in the context of Pakistan and India and other rapidly growing nations, as highlighted by Carvalho [60] and Sadigov [61], where the demand for food is projected to double in the coming decades [19,62]. Our findings corroborate the patterns reported by Sukereman *et al.* [55], who found that food security levels vary with income levels of countries. This study align with Thomas *et al.* [15] and Izraelov and Silber [8], who proposed that GFSI provides a comprehensive overview and it should be complemented with other indicators of food. Our study supports the multidimensional approach by illustrating how population growth alone does not determine food security but interacts with other determinates of food security, economic and policy variables etc. A key implication of our findings is that food security policy in highly populous countries must be proactive and systemic, integrating population control strategies with innovations in food production, storage and distribution. Additionally, international collaboration for technological support and investment in sustainable agriculture should be emphasized. This study argues the need for cross-national collaboration and adaptive food security policies. The findings of this study contribute to the ongoing debate on how best to utilize the GFSI as an indicator of food security, especially in the context of global population growth. The study also provides a useful reference point for future comparative research across different regions using the GFSI framework.

### Limitations and future research directions

While this study provides valuable insights into the relationship between GFSI and APC in the world’s five most populous countries, it is not without limitations. One of the key limitations of this study is the reliance on secondary data, which may contain inconsistencies or reporting biases across different countries. Another limitation stems from the study’s analytical approach. While correlation analysis, regression models and the Rule of Thumb (RoT) technique provide meaningful interpretations, but there may be establish definitive causal relationships between APC and GFSI using the supervised and unsupervised machine learning models. External factors such as economic policies, geopolitical instability, climate change, technological advancements in agriculture and productive aspects of population growth may also play crucial roles in shaping food security, yet these aspects are not directly incorporated into the model. Additionally, the study focuses on a limited time span (2012–2022), which may not fully capture the long term effects of APC on food security dynamics. Despite these limitations, this research lays a strong foundation for future studies, encouraging further exploration with expanded datasets, alternative modeling techniques, and deeper integration of socio-economic and environmental variables to enhance the applicability of food security strategies.

### recommendations and policy implications

The study suggests several policy implications to tackle the growing issue of global food insecurity which is driven by factors such as climate change, economic pressures, agricultural challenges, social dynamics and other global concerns. The focus is particularly on the five most populous countries (India, China, the USA, Indonesia and Pakistan). The following policy recommendations can be derived from the findings:

### Specific short term policy implications

- India must prioritize comprehensive measures across all sectors, with an immediate focus on improving its overall GFSI, particularly in AF and S&A. Proactive steps are needed to mitigate the negative impact of APC on GFSI, especially in the areas of Q&S and S&A.
- China’s overall performance is commendable. However, there is a need to further strengthen S&A. Targeted policies should be developed to mitigate the adverse impacts of APC on food security, with a particular focus on enhancing S&A.
- USA: The USA is effectively managing its GFSI. However, more focused efforts are required to reduce the impact of APC on Q&A as well as on S&A.
- Indonesia: Need to improve food security across all sectors with emphasizing on AV, Q&S and S&A. Mitigate the adverse effects of APC on the GFSI especially in the areas of AF and AV.
- Pakistan: Intensify efforts to boost its GFSI rank by enhancing performance across all sectors with a specific focus on implement population control measures to stabilize and to maintain the APC and reduce its negative impact on food security.

### Overall long term policy implications

- **Structural Population Management Strategies:** India, China, USA and Indonesia have successfully implemented population management strategies, keeping their growth within a tolerable reduction range. In contrast, Pakistan urgently needs to enforce effective population control measures to stabilize growth and sustain the reduction in APC to ensure long term food security. To achieve this, comprehensive long term policies must be developed, focusing on enhancing the productive potential of the population, mitigating the negative impact of population growth on food security. Strategic demographic policies, including education reforms and economic incentives should be introduced to regulate population growth effectively. Additionally, promoting a balanced rural-urban migration can optimize food distribution, ensuring equitable access to resources and sustainable development.
- **Promoting Global Cooperation for Sustainable Food Security:** Food security is a global challenge that requires collective efforts and international collaboration. By fostering partnerships and facilitating the exchange of knowledge and resources, it can be address effectively. It is essential to implement strategies that not only resolve immediate issues, but also enhance the long term resilience and sustainability of food systems. Additionally, recognizing the dynamic nature of food security, it must develop adaptable policies that can respond to shifting demographic trends, climate changes and other evolving factors. A proactive and cooperative approach will ensure a more secure and sustainable future for global food production and distribution. Improve international trade agreements to ensure food availability during periods of crisis. Facilitate collaboration between agricultural, economic and health sectors to integrate policy implementation. Encourage emergency response strategies to tackle food crises caused by climate change and economic disruptions.
- **Cross Sectorial Improvement and Statistical Significance:** All countries should strive for continuous improvements across all GFSI components by recognize the statistically significant and negative relation between GFSI and APC extracted from Rule of Thumbs.
- **Boost Agriculture Research:** Strengthen more agricultural research and development to sustain high levels of Food security. Increase investments in sustainable food production and climate smart agriculture and implement policies to further reduce food waste. Continue research and innovation in genetically modified crops to enhance resilience against climate change.

### Reproducibility and applicability of this work to other regions/countries

The study’s reproducibility and applicability are strong across various regions by conducting a CPA on the GFSI and population. The research methodology is adaptable to different national contexts and scales from global to local. The insights into how components like AF, AV, Q&S and S&A interact with APC offer a framework that can be applied to other regions. The study’s findings and recommendations provide a template that can guide policy interventions in diverse geographic and demographic settings enabling countries to tailor strategies that address their unique challenges in achieving food security.

## Conclusions

The pressing challenge of global food insecurity is fueled by population growth. It is imperative to guarantee universal access to safe, abundant and nutritious food. This research conducts a CPA on the GFSI scores, GFSI components (AF, AV, Q&S and S&A) and APC for the world’s five most populous countries i.e. India, China, USA, Indonesia and Pakistan using the datasets spinning from 2012–2022.

India’s GFSI rank is 68^th^. Positive advancements were observed for AV, Q&S and S&A while AF experienced a decline. APC found 0.68% in 2022 and showing the decreased of -49.25%. GFSI score has strong relation with AV (0.705), S&A (0.759) and Q&S (0.894) while moderate for AF (0.585). APC in relation with GFSI is effecting GFSI (-0.533) at moderate level, negligible for AF, low for AV (-0.299), and strong for Q&S (-0.658) and S&A (-0.874). It is recommended for India to work for improving its rank and to work on AF. China ranks good at 25^th^ position in GFSI rankings. The overall score improvements for China across sectors were impressive in AF, AV, Q&S, S&A and in APC. China has very strong relation of all sectors as AV (0.949), AF (0.804), Q&S (0.833) and S&A (0.906). Similarly APC in relation with GFSI has strong relation as -0.644, -0.750 and -0.651 for GFSI, AV and S&A. It is recommended for China to reduce it adverse impacts of APC on GFSI with reduction of its strong effect on AV and S&A. USA ranked good at 13^th^. GFSI score is slightly changed for all components. In spite of negative impact of APC, USA is well maintaining its GFSI but there is need to reduce the adverse impact of APC on Q&S and S&A. Indonesia stands at GFSI rank of 63^th^ in 2022. Indonesia witnesses very strong correlation for GFSI with AF (0.865), strong for AV (0.788) and S&A (0.620), and negligible for Q&S. In similar case, APC is adversary hitting food security showing the very strong relation for AF (-0.809), strong for GFSI (-0.643), AV (-0.606) and lower for S&A (-0.289). It is recommended for Indonesia to work for improving its rank and to work on all sectors with special emphasis on the reduction of APC and its adverse effect AF, AV and S&A. Pakistan stands at lower rank of 84^th^ in 2022. APC increases about 5.52%. For GFSI, Pakistan witnesses very strong correlation in AF and AV as 0.852 and 0.880, moderate for Q&S (-0.437), lower for S&A (0.251) and negligible for APC (-0.042). APC is adversary hitting at moderate level for AF (-0.485) while its effects lower for AV (0.347), negligible for Q&S (0.137) and strong for S&A (0.711). It is recommended for Pakistan to work more for improving its rank and to improve its all sector with the reduction of APC. This study has the potential to offer valuable policy recommendations aimed at improving food security by addressing key contributing factors.
